# P45 Analysing the impact of a novel microbiology MDT on endourology surgical outcomes in a complex tertiary centre

**DOI:** 10.1093/jacamr/dlag102.051

**Published:** 2026-06-26

**Authors:** Daniel Andreyev, Amelia Joseph, Hari Ratan

**Affiliations:** Nottingham University Hospitals, Nottingham, UK; Nottingham University Hospitals, Nottingham, UK; Nottingham University Hospitals, Nottingham, UK

## Abstract

**Background:**

Post-operative urinary infection following endourology surgery occurs in approximately 7%–10% of cases and is more common in prolonged or complex procedures.^1,2,3^ Infective complications may lead to significant morbidity, including prolonged hospitalization, readmission, or critical care admission. As a regional tertiary urology centre managing complex endourological referrals, the patient cohort at Nottingham Urology Centre is inherently high risk.

**Objectives:**

To mitigate this, we implemented a pioneering and novel microbiology-led multidisciplinary team (MDT) model, incorporating mandatory pre-operative urine culture and individualized antimicrobial prophylaxis. We aimed to evaluate post-operative infection outcomes following endourological interventions in our tertiary centre.

**Methods:**

A retrospective cohort study was conducted including 190 elective endourological patients in a 3-month period (Jan-Apr 2025). All patients underwent procedures with ureteric involvement; diagnostic or therapeutic rigid or flexible ureteroscopy (URS), percutaneous nephrolithotomy (PCNL), or ureteric stent exchange. We analysed surgical indication, waiting time for operation, ASA grading, whether patients had received interim procedures such as nephrostomy or emergency stent placement, urine microbiology results (including presence of multi-resistant pathogens), and postoperative infection rates (including critical care admission, prolonged hospital stay or readmission for infection). Cohort A comprised patients that experienced either prolonged hospital stays, or readmission within 14 days, for infection reasons. Cohort B included patients with no prolonged hospital stays or readmission within 14 days for infective symptoms.

**Results:**

Of 190 patients, the median age was 63, with an average wait time of 118.5 days. There were 31 Stent changes, 27 PCNLs, 5 TURBTs with URS and 127 URS. The median wait time for PCNL was 199 days and for URS 136 days. 74 patients had interim operations (either stent or nephrostomy) and 85 were primary cases. 69 patients (38%) required run in antibiotics prior to the operation with 121 requiring only prophylaxis on the day of operation. All patients had a pre-operative urine culture performed. 53% of patients had microbiologically significant growth of a uropathogen, of which 26.7% had multi-resistant organisms. 6.8% of patients were treated for UTI symptoms within 30 days of procedure. 4.2% of patients had a post-operative infection with either prolonged post-operative hospital stay, or readmission within 30 days with infection symptoms (Cohort A). The median length of prolonged stays and readmissions was 3.5 days. There were zero post-operative admissions to critical care in the 190 patients over 3 months. Only 50% of Cohort A had post-operative urine or blood cultures that were positive. 75% of Cohort A had significant growth on pre-operative urine culture compared to 52% of Cohort B. 62.5% of Cohort A required run in antibiotics compared to 35% in Cohort B, Cohort A were more likely to require run-in antibiotics (*P*=0.08). Cohort A had a significantly longer waiting time compared to Cohort B (*P*=0.049) (median 291 versus 115 days) and were likely to have a higher ASA grade (*P*=0.07).

**Conclusions:**

Our MDT approach to endourology surgery with mandatory urine culture and tailored antibiotic prophylaxis is associated with low post-operative infection rates, despite high clinical complexity and urine culture positivity. Despite over half of patients demonstrating significant pre-operative microbial growth and a substantial proportion harbouring multi-resistant organisms, infective complications prolonging hospital stay or causing readmission occurred in only 4.2% of cases, with no critical care admissions. Pre-operative urine culture positivity and longer waiting times were associated with the development of post-operative infective complications. These findings suggest that minimizing waiting times, particularly in complex cases, may further reduce post-operative infection risk. Our microbiology-integrated MDT model represents an effective model to deliver good infection outcomes in complex endourological patients.
